# Cardiovascular outcomes with potassium binders in patients with heart failure and hyperkalaemia

**DOI:** 10.1093/eschf/xvag248

**Published:** 2026-09-28

**Authors:** Laurent Fauchier, Mickael Guglieri, Thibault Lenormand, Thibaud Genet, Arnaud Bisson

**Affiliations:** Department of Cardiology, Centre Hospitalier Universitaire Trousseau et Faculté de Médecine, INSERM UMR1327 ISCHEMIA, Université François Rabelais, Avenue de la République, Tours 37044, France; Department of Cardiology, Centre Hospitalier Universitaire Trousseau et Faculté de Médecine, INSERM UMR1327 ISCHEMIA, Université François Rabelais, Avenue de la République, Tours 37044, France; Department of Cardiology, Centre Hospitalier Universitaire Trousseau et Faculté de Médecine, INSERM UMR1327 ISCHEMIA, Université François Rabelais, Avenue de la République, Tours 37044, France; Department of Cardiology, Centre Hospitalier Universitaire Trousseau et Faculté de Médecine, INSERM UMR1327 ISCHEMIA, Université François Rabelais, Avenue de la République, Tours 37044, France; Department of Cardiology, Centre Hospitalier Universitaire Trousseau et Faculté de Médecine, INSERM UMR1327 ISCHEMIA, Université François Rabelais, Avenue de la République, Tours 37044, France

**Keywords:** Heart failure, Hyperkalaemia, Potassium binders, Sodium zirconium cyclosilicate, Patiromer, Cardiovascular outcomes

## Abstract

**Background and Aims:**

Hyperkalaemia complicates heart failure (HF) and may limit guideline-directed therapy. Potassium binders differ in pharmacologic properties and sodium load, raising cardiovascular safety concerns. This study compared cardiovascular outcomes associated with patiromer, sodium zirconium cyclosilicate (SZC), and sodium polystyrene sulfonate (SPS).

**Methods:**

A retrospective observational study was conducted within the TriNetX network including adults with HF and hyperkalaemia initiating patiromer, SZC, or SPS (2019–2024). Three pairwise 1:1 propensity-matched comparisons were performed. Follow-up was up to 365 days (mean 0.7 ± 0.4 years). Outcomes included death, HF hospitalization, cardiovascular hospitalization, myocardial infarction, incident atrial fibrillation (AF), pulmonary oedema/cardiogenic shock, end-stage kidney disease (ESKD), and ventricular tachyarrhythmia/cardiac arrest.

**Results:**

After matching, 5589 patients per group were included for patiromer vs SPS, 15 769 per group for SZC vs SPS, and 4381 per group for SZC vs patiromer. Compared with SPS, patiromer was associated with lower risk of HF hospitalization (hazard ratio [HR] 0.573, 95% confidence interval [CI] 0.535–0.613) and cardiovascular hospitalization (HR 0.737, 95% CI 0.697–0.779), but slightly higher mortality (HR 1.096, 95% CI 1.027–1.170). SZC was associated with higher risks of death (HR 1.139, 95% CI 1.095–1.185), HF hospitalization (HR 1.184, 95% CI 1.143–1.225), myocardial infarction (HR 1.132, 95% CI 1.018–1.260), ESKD (HR 1.137, 95% CI 1.082–1.196), and cardiovascular hospitalization (HR 1.193, 95% CI 1.157–1.229) compared with SPS. SZC was associated with higher HF hospitalization (HR 2.735, 95% CI 2.519–2.969) and cardiovascular hospitalization (HR 1.914, 95% CI 1.797–2.037) than patiromer, with no significant difference in mortality.

**Conclusions:**

In this observational study of HF with hyperkalaemia, potassium binders were associated with different cardiovascular outcomes. These findings are hypothesis-generating and support randomized trials powered for clinical endpoints.

## Graphical Abstract

**Graphical Abstract:**
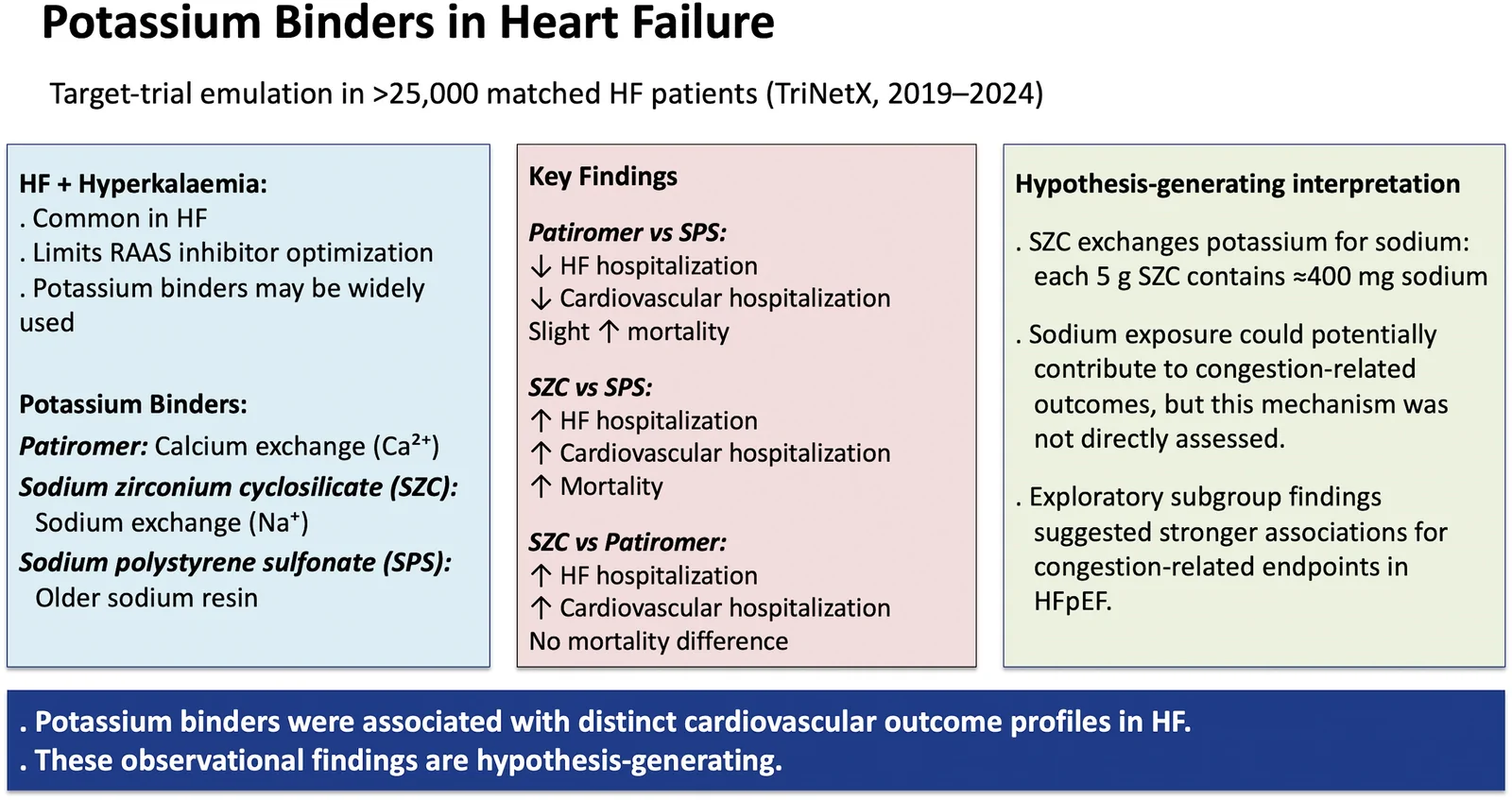
Cardiovascular Risk Profiles of Potassium Binders in Heart Failure This triple target-trial emulation in more than 25 000 propensity-matched patients with heart failure (HF) and hyperkalaemia compared sodium zirconium cyclosilicate (SZC), patiromer, and sodium polystyrene sulfonate (SPS). Patiromer was associated with lower risks of HF and cardiovascular hospitalization compared with SPS. In contrast, SZC was associated with higher risks of HF and cardiovascular hospitalization compared with both SPS and patiromer, without a mortality difference vs patiromer. The differential exchange ions—calcium for patiromer vs sodium for SZC and SPS—may have haemodynamic implications. Sodium load could contribute to volume expansion and increased filling pressures, particularly in HF with preserved ejection fraction.

## Introduction

Hyperkalaemia is a frequent and clinically consequential complication in patients with heart failure (HF), particularly in the context of chronic kidney disease, diabetes, and the use of renin–angiotensin–aldosterone system inhibitors (RAASi). Both hypo- and hyperkalaemia are associated with adverse cardiovascular outcomes, including arrhythmias and mortality, and dyskalaemia often leads to down-titration or discontinuation of guideline-directed medical therapy (GDMT).^1^ In contemporary HF registries, up to one-third of high-risk patients experience hyperkalaemic events, with consequent suboptimal RAASi and mineralocorticoid receptor antagonist use.^2^

Novel potassium binders, including patiromer and sodium zirconium cyclosilicate (SZC), have expanded therapeutic options and may facilitate GDMT optimization. However, these agents differ in their exchange ions (calcium vs sodium), raising concerns regarding potential fluid-related and cardiovascular effects. Observational studies have suggested a numerically or significantly higher risk of HF hospitalization with SZC compared with patiromer ,^3,4^ although randomized trials were not designed to assess hard cardiovascular outcomes.^5^ Sodium polystyrene sulfonate (SPS, Kayexalate), an older sodium-based resin, remains widely used despite limited contemporary comparative data and recognized safety limitations.

In this context, real-world comparative evidence on cardiovascular outcomes associated with different potassium binders in HF is limited. Leveraging the large, well-characterized TriNetX network, we conducted a comparative effectiveness study evaluating the risk of cardiovascular outcomes among patients with HF treated with SZC, patiromer, or SPS for hyperkalaemia in routine clinical practice.

## Methods

### Study design and data source

We conducted a retrospective comparative effectiveness study designed to emulate a target trial comparing cardiovascular outcomes among patients with HF and hyperkalaemia initiating SZC, patiromer, or SPS. The target-trial emulation framework is an observational approach that seeks to replicate the design and analysis of a hypothetical randomized trial using real-world data. The protocol components of the hypothetical target trial—including eligibility criteria, treatment strategies, assignment procedures, start of follow-up, and outcome definitions—were explicitly specified before analysis. This study was conducted using the TriNetX Global Collaborative Network, a federated research platform providing access to de-identified electronic medical records from more than 160 million patients and over 170 healthcare organizations worldwide. The database includes demographics, diagnoses (ICD-10-CM), procedures (CPT), medications (RxNorm), and laboratory measurements. Data are updated in real time, and analyses are performed within the secure TriNetX environment, with only aggregate results exported.

#### Study population

We identified adult patients aged 18 to 90 years with a diagnosis of HF (ICD-10-CM I50) and hyperkalaemia (ICD-10-CM E87.5) who initiated treatment with patiromer, SZC, or SPS (Kayexalate) between 1 January 2019 and 31 December 2024. To enhance diagnostic specificity and ensure clinical relevance, cohort inclusion required documentation of hyperkalaemia accompanied by a recorded potassium laboratory value, a diagnosis of HF, background cardiovascular medication use, and an available estimated glomerular filtration rate (eGFR) measurement. HF phenotype (HFrEF vs HFpEF) was defined using ICD-10 coding (I50.2 vs I50.3), as LVEF data were not consistently available across the cohort.

The index date was defined as the first qualifying prescription of the respective potassium binder meeting all inclusion criteria. To construct mutually exclusive exposure groups, patients receiving alternative potassium binders at baseline were excluded from comparator cohorts. Individuals with prior renal transplantation, dialysis procedures, or kidney transplant status were also excluded to focus on non–end-stage kidney disease populations.

Outcomes were assessed beginning one day after the index date to avoid inclusion of events occurring on the day of treatment initiation and to reduce temporal misclassification. Patients were followed for up to 365 days or until death, last recorded healthcare encounter, or end of data availability, whichever occurred first. Censoring occurred at the time of last recorded healthcare activity within the database.

Because potassium binders may be prescribed either as short-term therapy or for longer-term maintenance, we performed a pre-specified sensitivity analysis truncating follow-up at 6 months to evaluate the consistency of associations during early follow-up.

### Study outcomes

Outcomes were defined using standardized ICD-10 diagnostic and procedural codes within the TriNetX platform. All-cause mortality was identified from vital status records. Hospitalization for HF was defined by a primary diagnosis of HF (ICD-10 I50). Pulmonary oedema or cardiogenic shock was defined using ICD-10 codes J81 and R57.0. Ischaemic stroke or thromboembolism was defined using ICD-10 codes I63 and I74. Incident MI was defined using ICD-10 code I21. AF was defined using ICD-10 code I48. For incident AF and myocardial infarction, patients with a prior history of the respective outcome before the index date were excluded from those analyses. End-stage kidney disease or initiation of dialysis (ESKD) was defined using ICD-10 code N18.6 or dialysis procedures. Ventricular arrhythmias or cardiac arrest were defined using ICD-10 codes I47.2, I47.21, I49.0, and I46. Cardiovascular-related hospitalization was defined as hospitalization with a primary cardiovascular diagnosis (including HF, MI, stroke, AF, and conduction disorders). All baseline characteristics and outcome definitions, including ICD-10, CPT, and ATC codes, are detailed in the Supplementary Table S1.

### Statistical analysis

To minimize confounding, 1:1 propensity score matching was performed using a nearest-neighbour algorithm with a calliper width of 0.1 pooled standard deviations. Matching incorporated 77 baseline characteristics encompassing demographics, cardiovascular comorbidities, renal disease, diabetes, pulmonary disease, prior thromboembolic events, device implantation, medication use (including beta-blockers, ACE inhibitors or angiotensin receptor blockers, angiotensin receptor–neprilysin inhibitors, mineralocorticoid receptor antagonists, diuretics, statins, SGLT2 inhibitors, antiplatelet agents, and antiarrhythmic drugs), and laboratory parameters such as lipid profile, glycated haemoglobin, body mass index, blood pressure, and eGFR. Covariate balance after matching was assessed using standardized mean differences, with values below 0.10 considered indicative of adequate balance.

Time-to-event outcomes were analysed using Kaplan–Meier methods and Cox proportional hazards models, reporting hazard ratios (HRs) with 95% confidence intervals (CIs). Proportional hazards assumptions were evaluated using Schoenfeld residuals. When proportionality was violated, complementary relative risk (RR) estimates were examined. All analyses were conducted within the TriNetX analytics framework.

### Ethics

The study used de-identified data and was deemed exempt from institutional review board oversight by the TriNetX Ethics Committee. The study complied with the Declaration of Helsinki.

## Results

The cohort selection flow is presented in *Figure 1*. After application of inclusion and exclusion criteria, three pairwise matched populations were constructed to compare patiromer, SZC, and SPS.

**Figure 1:**
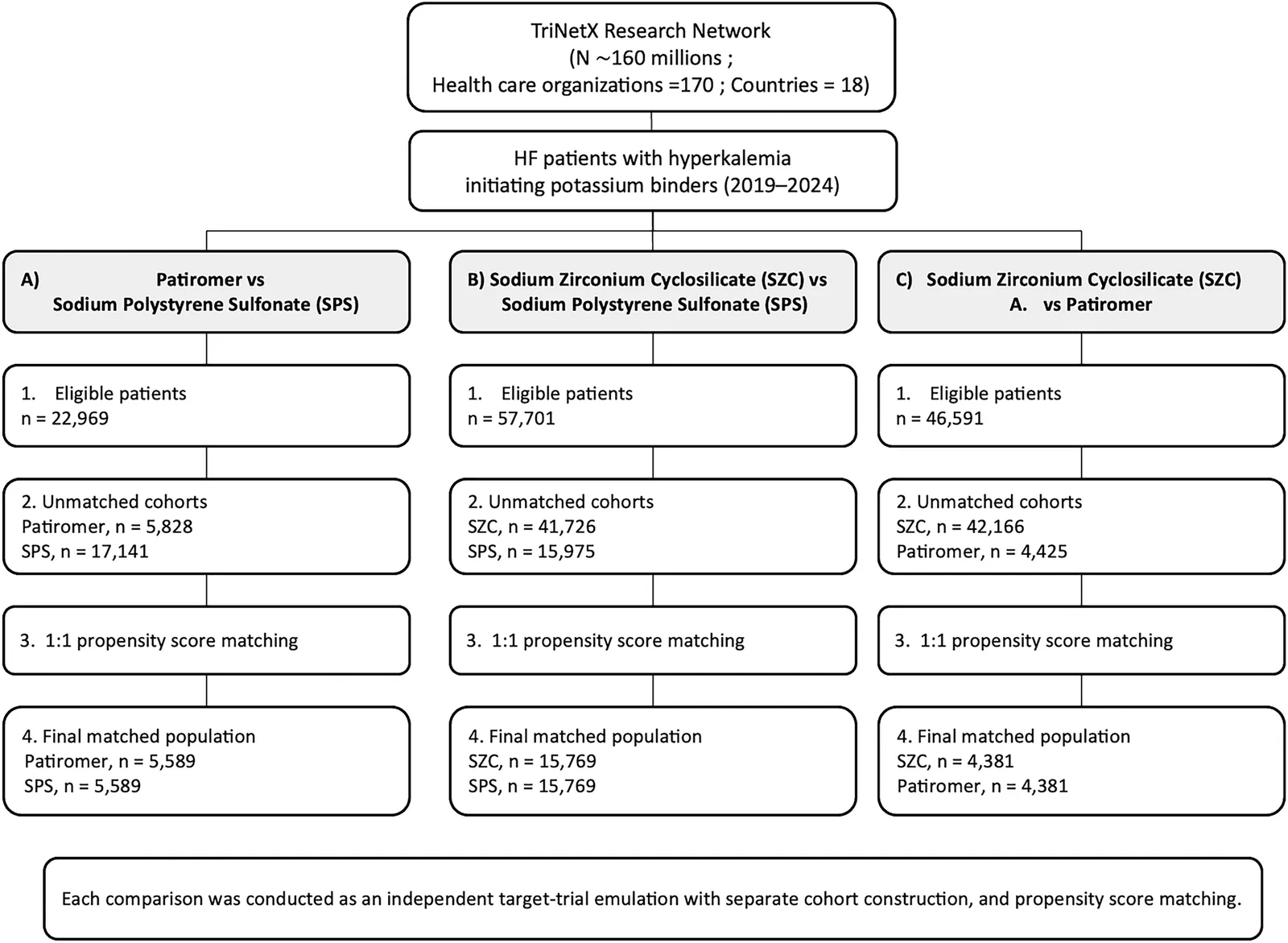
Flow chart of patients in the study

For the comparison of patiromer vs SPS, 5589 patients were included in each group after 1:1 propensity score matching. For SZC vs SPS, 15 769 patients were matched in each cohort. For SZC vs patiromer, 4381 patients were included per group. Patients were elderly (mean age ∼70–71 years), predominantly male (∼55%), with a high burden of comorbidities including chronic kidney disease (>93%), diabetes (∼65%), AF (∼50%), and coronary artery disease (∼60%). Background heart failure therapy was widely used, including beta-blockers (∼83%), renin–angiotensin system inhibitors, mineralocorticoid receptor antagonists (∼30%), diuretics (∼90%), and sodium–glucose cotransporter-2 inhibitors in ∼10%). Across all matched comparisons, covariate balance was excellent, with standardized mean differences below 0.10 for all baseline categorical characteristics and below 0.12 for all numerical variables (*Tables 1–3*). Available LVEF values were similar across groups after matching, although measurements were only available in a subset of patients.

**Table 1:** Baseline characteristics before and after propensity score matching for HF patients treated with patiromer or sodium polystyrene sulfonate

	Before propensity-score matching			After propensity-score matching		
	Patiromer	Sodium polystyrene sulfonate	Std diff. (%)	Patiromer	Sodium polystyrene sulfonate	Std diff. (%)
	(*n* = 5828)	(*n* = 17 141)		(*n* = 5589)	(*n* = 5589)	
*Demographics*						
Age (years), mean ± SD	71.0 +/− 13.1	70.8 +/− 13.2	1.5	71.0 +/− 13.0	71.1 +/− 13.2	0.7
Men, *n* (%)	3200 (54.9%)	9057 (52.8%)	4.2	3055 (54.7%)	3006 (53.8%)	1.8
White, *n* (%)	3849 (66%)	11 281 (65.8%)	0.5	3673 (65.7%)	3671 (65.7%)	0.1
Black or African American, *n* (%)	1374 (23.6%)	3850 (22.5%)	2.6	1319 (23.6%)	1309 (23.4%)	0.4
Asian, *n* (%)	167 (2.9%)	626 (3.7%)	4.4	166 (3%)	186 (3.3%)	2
Hispanic or Latino, *n* (%)	252 (4.3%)	869 (5.1%)	3.5	248 (4.4%)	254 (4.5%)	0.5
Socioeconomic and psychosocial circumstances, *n* (%)	410 (7%)	1046 (6.1%)	3.8	371 (6.6%)	364 (6.5%)	0.5
*Risk factors*						
Hypertension, *n* (%)	4518 (77.5%)	13 459 (78.5%)	2.4	4315 (77.2%)	4348 (77.8%)	1.4
Diabetes mellitus, *n* (%)	3778 (64.8%)	11 156 (65.1%)	0.5	3611 (64.6%)	3632 (65%)	0.8
Smoker, *n* (%)	2581 (44.3%)	7589 (44.3%)	0	2444 (43.7%)	2431 (43.5%)	0.5
Overweight or obesity, *n* (%)	2588 (44.4%)	7570 (44.2%)	0.5	2475 (44.3%)	2498 (44.7%)	0.8
Dyslipidaemia, *n* (%)	4431 (76%)	12 893 (75.2%)	1.9	4223 (75.6%)	4222 (75.5%)	0
Alcohol-related diagnoses, *n* (%)	343 (5.9%)	1050 (6.1%)	1	334 (6%)	325 (5.8%)	0.7
*Cardiovascular comorbidities*						
Coronary artery disease, *n* (%)	3466 (59.5%)	10 715 (62.5%)	6.2	3329 (59.6%)	3349 (59.9%)	0.7
Myocardial infarction, *n* (%)	1469 (25.2%)	4737 (27.6%)	5.5	1405 (25.1%)	1402 (25.1%)	0.1
Ischaemic stroke, *n* (%)	689 (11.8%)	2095 (12.2%)	1.2	649 (11.6%)	661 (11.8%)	0.7
Intracranial haemorrhage, *n* (%)	78 (1.3%)	247 (1.4%)	0.9	74 (1.3%)	64 (1.1%)	1.6
Valve disease, *n* (%)	1270 (21.8%)	4516 (26.3%)	10.7	1236 (22.1%)	1281 (22.9%)	1.9
Atrial fibrillation or flutter, *n* (%)	2831 (48.6%)	8467 (49.4%)	1.6	2689 (48.1%)	2693 (48.2%)	0.1
*Non-cardiovascular comorbidities*						
Kidney disease, *n* (%)	5496 (94.3%)	15 721 (91.7%)	10.2	5261 (94.1%)	5269 (94.3%)	0.6
COPD, *n* (%)	2019 (34.6%)	6404 (37.4%)	5.7	1950 (34.9%)	1938 (34.7%)	0.5
Sleep apnoea syndrome, *n* (%)	1875 (32.2%)	5058 (29.5%)	5.8	1771 (31.7%)	1747 (31.3%)	0.9
Peripheral vascular disease, *n* (%)	1098 (18.8%)	3334 (19.5%)	1.6	1050 (18.8%)	1055 (18.9%)	0.2
Previous neoplasm, *n* (%)	1948 (33.4%)	5515 (32.2%)	2.7	1842 (33%)	1884 (33.7%)	1.6
Cognitive impairment, *n* (%)	113 (1.9%)	391 (2.3%)	2.4	107 (1.9%)	103 (1.8%)	0.5
*Laboratory tests and examinations*						
Systolic BP (mm Hg), mean ± SD	124.0 +/− 23.7	122.2 +/− 24.3	7.5	124.3 +/− 23.7	123.9 +/− 23.7	1.3
Diastolic BP (mm Hg), mean ± SD	66.8 +/− 14.2	65.4 +/− 14.8	9.1	66.8 +/− 14.2	66.1 +/− 14.4	5.1
Heart rate, mean ± SD	78.3 +/− 17.6	78.4 +/− 17.9	0.8	78.5 +/− 17.8	77.7 +/− 17.6	4.3
Body mass index (kg/m2), mean ± SD	31.5 +/− 9.3	31.1 +/− 9.5	4.4	31.5 +/− 9.3	30.9 +/− 9.4	6.1
Total cholesterol (mg/dL), mean ± SD	144.0 +/− 46.4	144.1 +/− 48.0	0.2	144.1 +/− 46.3	143.3 +/− 47.0	1.7
Estimated GFR (MDRD, ml/min), mean ± SD	29.8 +/− 21.6	32.2 +/− 23.9	10.6	29.9 +/− 21.8	30.2 +/− 22.5	1.3
Haemoglobin A1c (%), mean ± SD	6.8 +/− 1.7	7.0 +/− 1.9	6.8	6.8 +/− 1.7	6.9 +/− 1.9	3.2
BNP, ng/L, mean ± SD	1096.9 +/− 2331.7	1098.9 +/− 2615.6	0.1	1093.1 +/− 2340.0	1126.3 +/− 2384.8	1.4
NT-proBNP, ng/L, mean ± SD	8236.8 +/− 11450.8	8070.1 +/− 11679.8	1.4	8169.6 +/− 11356.1	8011.5 +/− 11596.8	1.4
LVEF, mean ± SD	47.1 +/− 17.7	49.3 +/− 17.1	12.6	47.1 +/− 17.8	47.7 +/− 14.8	3.8
Potassium (mEq/L), mean ± SD	5.3 +/− 0.6	5.3 +/− 0.7	1.5	5.3 +/− 0.6	5.3 +/− 0.7	1.3
Potassium (mEq/L), median (IQR)	5.3 (0.6)	5.3 (0.8)	9.3	5.3 (0.6)	5.3 (0.8)	2.1
Potassium > 6.0 mEq/L, *n* (%)	2497 (42.8%)	6924 (40.4%)	5	2359 (42.2%)	2380 (42.6%)	0.8
*Medications*						
Beta Blockers, *n* (%)	4861 (83.4%)	14 167 (82.6%)	2	4638 (83%)	4670 (83.6%)	1.5
Calcium Channel Blockers, *n* (%)	3074 (52.7%)	9501 (55.4%)	5.4	2969 (53.1%)	3027 (54.2%)	2.1
ACE inhibitors, *n* (%)	2160 (37.1%)	7048 (41.1%)	8.3	2095 (37.5%)	2092 (37.4%)	0.1
Angiotensin II inhibitors, *n* (%)	1814 (31.1%)	5285 (30.8%)	0.6	1707 (30.5%)	1782 (31.9%)	2.9
ARNI, *n* (%)	591 (10.1%)	1289 (7.5%)	9.2	517 (9.3%)	546 (9.8%)	1.8
MRA, *n* (%)	1773 (30.4%)	4908 (28.6%)	3.9	1653 (29.6%)	1693 (30.3%)	1.6
SGLT2 inhibitors, *n* (%)	603 (10.3%)	1061 (6.2%)	15.1	496 (8.9%)	512 (9.2%)	1
Diuretics, *n* (%)	5226 (89.7%)	15 123 (88.2%)	4.6	4989 (89.3%)	5024 (89.9%)	2
Digitalis glycosides, *n* (%)	537 (9.2%)	1702 (9.9%)	2.4	505 (9%)	525 (9.4%)	1.2
Amiodarone, *n* (%)	827 (14.2%)	2661 (15.5%)	3.8	779 (13.9%)	766 (13.7%)	0.7
Lipid lowering drugs, *n* (%)	4302 (73.8%)	12 455 (72.7%)	2.6	4103 (73.4%)	4100 (73.4%)	0.1
Glucose-lowering therapy, *n* (%)	4795 (82.3%)	14 023 (81.8%)	1.2	4574 (81.8%)	4588 (82.1%)	0.7
Metformin, *n* (%)	966 (16.6%)	3216 (18.8%)	5.7	923 (16.5%)	928 (16.6%)	0.2
Long-term use of insulin, *n* (%)	2041 (35%)	5317 (31%)	8.5	1912 (34.2%)	1888 (33.8%)	0.9
Antiplatelet therapy, *n* (%)	4037 (69.3%)	12 336 (72%)	5.9	3879 (69.4%)	3911 (70%)	1.2
Anticoagulant, *n* (%)	2114 (36.3%)	5470 (31.9%)	9.2	1972 (35.3%)	1968 (35.2%)	0.1

Values are *n* (%) or mean ± SD.

ACE, angiotensin converting enzyme; AF, atrial fibrillation; ARNI, Angiotensin Receptor–Neprilysin Inhibitor; BNP, Brain Natriuretic Peptide; BP, blood pressure; COPD, Chronic obstructive pulmonary disease; eGFR, estimated glomerular filtration rate; LVEF, left ventricular ejection fraction; MRA, Mineralocorticoid Receptor Antagonist; NT-proBNP, N-Terminal Prohormone Brain Natriuretic Peptide; SD, standard deviation.

**Table 2:** Baseline characteristics before and after propensity score matching for HF patients treated with sodium zirconium or sodium polystyrene sulfonate

	Before propensity-score matching			After propensity-score matching		
	Sodium zirconium	Sodium polystyrene sulfonate	Std diff. (%)	Sodium zirconium	Sodium polystyrene sulfonate	Std diff. (%)
	(*n* = 41 726)	(*n* = 15 975)		(*n* = 15 769)	(*n* = 15 769)	
*Demographics*						
Age (years), mean ± SD	70.7 +/− 13.5	70.3 +/− 13.5	2.5	70.3 +/− 13.5	70.4 +/− 13.5	0.4
Men, *n* (%)	22 765 (54.6%)	8631 (54%)	1.1	8549 (54.2%)	8498 (53.9%)	0.6
White, *n* (%)	24 032 (57.6%)	9560 (59.8%)	4.6	9415 (59.7%)	9415 (59.7%)	0
Black or African American, *n* (%)	11 583 (27.8%)	4340 (27.2%)	1.3	4306 (27.3%)	4292 (27.2%)	0.2
Asian, *n* (%)	2001 (4.8%)	682 (4.3%)	2.5	652 (4.1%)	678 (4.3%)	0.8
Hispanic or Latino, *n* (%)	2534 (6.1%)	883 (5.5%)	2.3	869 (5.5%)	875 (5.5%)	0.2
Socioeconomic and psychosocial circumstances, *n* (%)	4319 (10.4%)	991 (6.2%)	15.1	996 (6.3%)	991 (6.3%)	0.1
*Risk factors*						
Hypertension, *n* (%)	32 974 (79%)	12 581 (78.8%)	0.7	12 399 (78.6%)	12 417 (78.7%)	0.3
Diabetes mellitus, *n* (%)	27 025 (64.8%)	10 413 (65.2%)	0.9	10 319 (65.4%)	10 257 (65%)	0.8
Smoker, *n* (%)	17 208 (41.2%)	6879 (43.1%)	3.7	6754 (42.8%)	6758 (42.9%)	0.1
Overweight or obesity, *n* (%)	18 604 (44.6%)	7014 (43.9%)	1.4	7003 (44.4%)	6926 (43.9%)	1
Dyslipidaemia, *n* (%)	32 683 (78.3%)	11 945 (74.8%)	8.4	11 847 (75.1%)	11 811 (74.9%)	0.5
Alcohol related diagnoses, *n* (%)	2757 (6.6%)	1043 (6.5%)	0.3	1003 (6.4%)	1029 (6.5%)	0.7
*Cardiovascular comorbidities*						
Coronary artery disease, *n* (%)	26 206 (62.8%)	9840 (61.6%)	2.5	9748 (61.8%)	9697 (61.5%)	0.7
Myocardial infarction, *n* (%)	12 531 (30%)	4411 (27.6%)	5.3	4322 (27.4%)	4374 (27.7%)	0.7
Ischaemic stroke, *n* (%)	5879 (14.1%)	2035 (12.7%)	4	2031 (12.9%)	2020 (12.8%)	0.2
Intracranial haemorrhage, *n* (%)	772 (1.9%)	246 (1.5%)	2.4	240 (1.5%)	244 (1.5%)	0.2
Valve disease, *n* (%)	10 880 (26.1%)	4139 (25.9%)	0.4	4112 (26.1%)	4069 (25.8%)	0.6
Atrial fibrillation or flutter, *n* (%)	20 462 (49%)	7750 (48.5%)	1.1	7638 (48.4%)	7635 (48.4%)	0
*Non-cardiovascular comorbidities*						
Kidney disease, *n* (%)	39 087 (93.7%)	14 827 (92.8%)	3.4	14 689 (93.2%)	14 650 (92.9%)	1
COPD, *n* (%)	13 423 (32.2%)	5586 (35%)	5.9	5487 (34.8%)	5500 (34.9%)	0.2
Sleep apnoea syndrome, *n* (%)	12 070 (28.9%)	4602 (28.8%)	0.3	4610 (29.2%)	4540 (28.8%)	1
Peripheral vascular disease, *n* (%)	8065 (19.3%)	3141 (19.7%)	0.8	3076 (19.5%)	3087 (19.6%)	0.2
Previous neoplasm, *n* (%)	13 932 (33.4%)	5252 (32.9%)	1.1	5077 (32.2%)	5183 (32.9%)	1.4
Cognitive impairment, *n* (%)	840 (2%)	357 (2.2%)	1.5	384 (2.4%)	352 (2.2%)	1.3
*Laboratory tests and examinations*						
Systolic BP (mm Hg), mean ± SD	122.0 +/− 25.3	122.2 +/− 25.0	0.9	122.0 +/− 25.1	122.3 +/− 25.0	1.1
Diastolic BP (mm Hg), mean ± SD	65.3 +/− 15.3	65.1 +/− 15.0	1.3	64.9 +/− 15.3	65.1 +/− 15.0	1.2
Heart rate, mean ± SD	79.3 +/− 19.4	78.8 +/− 18.7	2.7	79.0 +/− 19.4	78.8 +/− 18.7	1.1
Body mass index (kg/m^2^), mean ± SD	30.2 +/− 9.0	30.7 +/− 9.3	5.4	30.7 +/− 9.4	30.7 +/− 9.3	0.6
Total cholesterol (mg/dL), mean ± SD	143.6 +/− 48.4	143.8 +/− 48.4	0.4	144.6 +/− 49.7	143.7 +/− 48.4	1.8
Estimated GFR (MDRD, ml/min), mean ± SD	30.1 +/− 24.5	30.9 +/− 23.8	3.3	30.1 +/− 23.9	30.9 +/− 23.8	3.3
Haemoglobin A1c (%), mean ± SD	6.8 +/− 1.8	6.9 +/− 1.9	6.1	6.9 +/− 1.8	6.9 +/− 1.9	1.1
BNP, ng/L, mean ± SD	2168.6 +/− 5745.6	1232.1 +/− 3403.3	19.8	1418.7 +/− 3641.1	1238.8 +/− 3426.4	5.1
NT-proBNP, ng/L, mean ± SD	8966.5 +/− 12778.0	8718.3 +/− 12298.7	2	8813.4 +/− 12364.5	8746.1 +/− 12336.7	0.5
LVEF, mean ± SD	49.9 +/− 16.5	49.0 +/− 17.1	5.4	49.3 +/− 16.5	49.2 +/− 17.0	0.1
Potassium (mEq/L), mean ± SD	5.2 +/− 0.7	5.3 +/− 0.7	8	5.3 +/− 0.7	5.3 +/− 0.7	5.2
Potassium (mEq/L), median (IQR)	5.3 (0.7)	5.3 (0.8)	9.3	5.3 (0.7)	5.3 (0.8)	2.1
Potassium > 6.0 mEq/L, *n* (%)	15 723 (37.7%)	6344 (39.7%)	4.2	6203 (39.3%)	6219 (39.4%)	0.2
*Medications*						
Beta blockers, *n* (%)	33 580 (80.5%)	13 244 (82.9%)	6.3	13 044 (82.7%)	13 043 (82.7%)	0
Calcium channel blockers, *n* (%)	22 895 (54.9%)	9060 (56.7%)	3.7	8840 (56.1%)	8933 (56.6%)	1.2
ACE inhibitors, *n* (%)	13 574 (32.5%)	6606 (41.4%)	18.4	6405 (40.6%)	6435 (40.8%)	0.4
Angiotensin II inhibitors, *n* (%)	15 590 (37.4%)	4932 (30.9%)	13.7	4888 (31%)	4895 (31%)	0.1
ARNI, *n* (%)	4848 (11.6%)	1159 (7.3%)	15	1133 (7.2%)	1149 (7.3%)	0.4
MRA, *n* (%)	11 104 (26.6%)	4488 (28.1%)	3.3	4301 (27.3%)	4369 (27.7%)	1
SGLT2 inhibitors, *n* (%)	6841 (16.4%)	968 (6.1%)	33.2	946 (6%)	968 (6.1%)	0.6
Diuretics, *n* (%)	35 183 (84.3%)	14 004 (87.7%)	9.6	13 790 (87.5%)	13 800 (87.5%)	0.2
Digitalis glycosides, *n* (%)	2797 (6.7%)	1451 (9.1%)	8.8	1073 (6.8%)	1407 (8.9%)	7.9
Amiodarone, *n* (%)	6457 (15.5%)	2462 (15.4%)	0.2	2455 (15.6%)	2409 (15.3%)	0.8
Lipid-lowering drugs, *n* (%)	30 573 (73.3%)	11 653 (72.9%)	0.7	11 474 (72.8%)	11 492 (72.9%)	0.3
Glucose-lowering therapy, *n* (%)	33 035 (79.2%)	13 190 (82.6%)	8.6	13 037 (82.7%)	12 992 (82.4%)	0.8
Metformin, *n* (%)	7075 (17%)	2956 (18.5%)	4.1	2832 (18%)	2874 (18.2%)	0.7
Long-term use of insulin, *n* (%)	11 146 (26.7%)	4727 (29.6%)	6.4	4632 (29.4%)	4626 (29.3%)	0.1
Antiplatelet therapy, *n* (%)	27 153 (65.1%)	11 372 (71.2%)	13.1	11 126 (70.6%)	11 174 (70.9%)	0.7
Anticoagulant, *n* (%)	12 463 (29.9%)	4772 (29.9%)	0	4730 (30%)	4689 (29.7%)	0.6

Values are *n* (%) or mean ± SD.

ACE, angiotensin-converting enzyme; AF, atrial fibrillation; ARNI, angiotensin receptor–neprilysin inhibitor; BNP, brain natriuretic peptide; BP, blood pressure; COPD, chronic obstructive pulmonary disease; eGFR, estimated glomerular filtration rate; LVEF, left ventricular ejection fraction; MRA, mineralocorticoid receptor antagonist; NT-proBNP, N-terminal prohormone brain natriuretic peptide; SD, standard deviation.

**Table 3:** Baseline characteristics before and after propensity score matching for HF patients treated with sodium zirconium or patiromer

	Before propensity-score matching			After propensity-score matching		
	Sodium zirconium	Patiromer	Std diff. (%)	Sodium zirconium	Patiromer	Std diff. (%)
	(*n* = 42 166)	(*n* = 4425)		(*n* = 4381)	(*n* = 4381)	
*Demographics*						
Age (years), mean ± SD	70.6 +/− 13.5	71.2 +/− 13.2	4.1	71.4 +/− 13.6	71.2 +/− 13.2	1
Men, *n* (%)	23 000 (54.5%)	2451 (55.4%)	1.7	2450 (55.9%)	2420 (55.2%)	1.4
White, *n* (%)	24 227 (57.5%)	2957 (66.8%)	19.4	2979 (68%)	2918 (66.6%)	3
Black or African American, *n* (%)	11 819 (28%)	886 (20%)	18.8	851 (19.4%)	883 (20.2%)	1.8
Asian, *n* (%)	2002 (4.7%)	171 (3.9%)	4.4	153 (3.5%)	171 (3.9%)	2.2
Hispanic or Latino, *n* (%)	2534 (6%)	246 (5.6%)	1.9	244 (5.6%)	246 (5.6%)	0.2
Socioeconomic and psychosocial circumstances, *n* (%)	4326 (10.3%)	310 (7%)	11.6	296 (6.8%)	307 (7%)	1
*Risk factors*						
Hypertension, *n* (%)	33 274 (78.9%)	3645 (82.4%)	8.8	3646 (83.2%)	3602 (82.2%)	2.7
Diabetes mellitus, *n* (%)	27 227 (64.6%)	2820 (63.7%)	1.8	2826 (64.5%)	2781 (63.5%)	2.1
Smoker, *n* (%)	17 232 (40.9%)	1860 (42%)	2.4	1851 (42.3%)	1839 (42%)	0.6
Overweight or obesity, *n* (%)	18 708 (44.4%)	1873 (42.3%)	4.1	1785 (40.7%)	1852 (42.3%)	3.1
Dyslipidaemia, *n* (%)	32 861 (77.9%)	3405 (76.9%)	2.4	3376 (77.1%)	3367 (76.9%)	0.5
Alcohol related diagnoses, *n* (%)	2767 (6.6%)	245 (5.5%)	4.3	241 (5.5%)	244 (5.6%)	0.3
*Cardiovascular comorbidities*						
Coronary artery disease, *n* (%)	26 340 (62.5%)	2634 (59.5%)	6	2612 (59.6%)	2607 (59.5%)	0.2
Myocardial infarction, *n* (%)	12 595 (29.9%)	1185 (26.8%)	6.9	1173 (26.8%)	1178 (26.9%)	0.3
Ischaemic stroke, *n* (%)	5916 (14%)	554 (12.5%)	4.5	539 (12.3%)	547 (12.5%)	0.6
Intracranial haemorrhage, *n* (%)	775 (1.8%)	60 (1.4%)	3.8	58 (1.3%)	60 (1.4%)	0.4
Valve disease, *n* (%)	10 918 (25.9%)	1000 (22.6%)	7.7	1044 (23.8%)	994 (22.7%)	2.7
Atrial fibrillation or flutter, *n* (%)	20 603 (48.9%)	2201 (49.7%)	1.8	2216 (50.6%)	2173 (49.6%)	2
*Non-cardiovascular comorbidities*						
Kidney disease, *n* (%)	39 424 (93.5%)	4158 (94%)	1.9	4113 (93.9%)	4114 (93.9%)	0.1
COPD, *n* (%)	13 489 (32%)	1416 (32%)	0	1404 (32%)	1400 (32%)	0.2
Sleep apnoea syndrome, *n* (%)	12 131 (28.8%)	1345 (30.4%)	3.6	1323 (30.2%)	1324 (30.2%)	0
Peripheral vascular disease, *n* (%)	8124 (19.3%)	853 (19.3%)	0	857 (19.6%)	842 (19.2%)	0.9
Previous neoplasm, *n* (%)	14 010 (33.2%)	1558 (35.2%)	4.2	1550 (35.4%)	1543 (35.2%)	0.3
Cognitive impairment, *n* (%)	845 (2%)	66 (1.5%)	3.9	64 (1.5%)	65 (1.5%)	0.2
*Laboratory tests and examinations*						
Systolic BP (mm Hg), mean ± SD	122.0 +/− 25.3	123.0 +/− 24.5	3.8	122.0 +/− 24.5	123.1 +/− 24.5	4.5
Diastolic BP (mm Hg), mean ± SD	65.3 +/− 15.3	66.0 +/− 14.4	4.2	64.8 +/− 14.5	65.9 +/− 14.4	8
Heart rate, mean ± SD	79.3 +/− 19.4	78.3 +/− 18.3	5.5	78.0 +/− 18.6	78.4 +/− 18.4	1.7
Body mass index (kg/m^2^), mean ± SD	30.2 +/− 9.0	31.2 +/− 9.2	11	30.2 +/− 8.8	31.1 +/− 9.2	10.1
Total cholesterol (mg/dL), mean ± SD	143.6 +/− 48.4	141.8 +/− 44.5	3.9	142.7 +/− 46.7	142.0 +/− 44.5	1.5
Estimated GFR (MDRD, ml/min), mean ± SD	30.1 +/− 24.4	28.3 +/− 21.3	7.9	28.3 +/− 22.3	28.3 +/− 21.3	0.1
Haemoglobin A1c (%), mean ± SD	6.8 +/− 1.8	6.8 +/− 1.7	0.6	6.9 +/− 1.8	6.8 +/− 1.7	5.9
BNP, ng/L, mean ± SD	2155.5 +/− 5707.7	1780.6 +/− 4814.2	7.1	2344.1 +/− 5998.1	1781.3 +/− 4815.9	10.3
NT-proBNP, ng/L, mean ± SD	8976.5 +/− 12789.2	8588.4 +/− 11877.5	3.1	8762.3 +/− 12121.8	8584.9 +/− 11821.9	1.5
LVEF, mean ± SD	49.9 +/− 16.5	47.1 +/− 16.5	17	48.5 +/− 14.1	47.1 +/− 16.5	8.8
Potassium (mEq/L), mean ± SD	5.2 +/− 0.7	5.3 +/− 0.6	9.3	5.3 +/− 0.6	5.3 +/− 0.6	2.1
Potassium (mEq/L), median (IQR)	5.3 (0.7)	5.3 (0.6)	9.3	5.3 (0.8)	5.3 (0.6)	2.1
Potassium > 6.0 mEq/L, *n* (%)	15 875 (37.6%)	1858 (42%)	8.9	1846 (42.1%)	1823 (41.6%)	1.1
*Medications*						
Beta blockers, *n* (%)	33 924 (80.5%)	3691 (83.4%)	7.7	3622 (82.7%)	3647 (83.2%)	1.5
Calcium channel blockers, *n* (%)	23 141 (54.9%)	2348 (53.1%)	3.6	2334 (53.3%)	2330 (53.2%)	0.2
ACE inhibitors, *n* (%)	13 699 (32.5%)	1735 (39.2%)	14	1733 (39.6%)	1708 (39%)	1.2
Angiotensin II inhibitors, *n* (%)	15 782 (37.4%)	1425 (32.2%)	11	1392 (31.8%)	1414 (32.3%)	1.1
ARNI, *n* (%)	4923 (11.7%)	460 (10.4%)	4.1	445 (10.2%)	455 (10.4%)	0.8
MRA, *n* (%)	11 232 (26.6%)	1356 (30.6%)	8.9	1338 (30.5%)	1331 (30.4%)	0.3
SGLT2 inhibitors, *n* (%)	6871 (16.3%)	536 (12.1%)	12	535 (12.2%)	535 (12.2%)	0
Diuretics, *n* (%)	35 543 (84.3%)	3990 (90.2%)	17.7	3943 (90%)	3946 (90.1%)	0.2
Digitalis glycosides, *n* (%)	2834 (6.7%)	382 (8.6%)	7.2	301 (6.9%)	372 (8.5%)	6.1
Amiodarone, *n* (%)	6520 (15.5%)	589 (13.3%)	6.1	584 (13.3%)	580 (13.2%)	0.3
Lipid-lowering drugs, *n* (%)	30 881 (73.2%)	3332 (75.3%)	4.7	3338 (76.2%)	3291 (75.1%)	2.5
Glucose-lowering therapy, *n* (%)	33 378 (79.2%)	3628 (82%)	7.2	3612 (82.4%)	3588 (81.9%)	1.4
Metformin, *n* (%)	7118 (16.9%)	802 (18.1%)	3.3	815 (18.6%)	792 (18.1%)	1.4
Long-term use of insulin, *n* (%)	11 245 (26.7%)	1441 (32.6%)	12.9	1464 (33.4%)	1406 (32.1%)	2.8
Antiplatelet therapy, *n* (%)	27 445 (65.1%)	3030 (68.5%)	7.2	3015 (68.8%)	2992 (68.3%)	1.1
Anticoagulant, *n* (%)	12 522 (29.7%)	1530 (34.6%)	10.5	1482 (33.8%)	1497 (34.2%)	0.7

Values are *n* (%) or mean ± SD.

ACE, angiotensin converting enzyme; AF, atrial fibrillation; ARNI, angiotensin receptor–neprilysin inhibitor; BNP, brain natriuretic peptide; BP, blood pressure; COPD, chronic obstructive pulmonary disease; eGFR, estimated glomerular filtration rate; LVEF, left ventricular ejection fraction; MRA, mineralocorticoid receptor antagonist; NT-proBNP, N-terminal prohormone brain natriuretic peptide; SD, standard deviation.

### Patiromer vs sodium polystyrene sulfonate

During a mean follow-up of 0.7 ± 0.4 years (with a median of 1.0 year reflecting administrative censoring), patiromer use was associated with a significantly lower risk of hospitalization for HF compared with SPS (26.21% vs 42.43% yearly rate; RR 0.63, 95% CI 0.595–0.668; *P* < .0001). Risk of cardiovascular-related hospitalization was also significantly lower (47.01% vs 57.18%; RR 0.816, 95% CI 0.783–0.850; *P* < .0001). In contrast, patiromer was associated with a modestly higher risk of all-cause mortality (37.05% vs 34.87%; RR 1.086, 95% CI 1.030–1.146; *P* = .002) and pulmonary oedema or cardiogenic shock (HR 1.224, 95% CI 1.114–1.345; *P* < .0001). Risks of incident myocardial infarction, atrial fibrillation, ventricular tachyarrhythmia or cardiac arrest, and end-stage kidney disease did not differ significantly between groups (*Table 4*).

**Table 4:** Clinical outcomes during FU in the matched populations (follow-up 0.7 ± 0.4 years, median 1, IQR 0.8)

	Patiromer		Sodium polystyrene sulfonate			
	(*n* = 5589)		(*n* = 5589)			
	Number of events	Yearly rate, %	Number of events	Yearly rate, %	Hazard ratio (95% CI)	*P* value
Death	1887	37.05	1737	34.87	1.086 (1.030–1.146)^a^	.002
Incident MI	264	9.37	245	8.61	1.076 (0.905–1.281)	.41
Hospitalization for HF	1325	26.21	2103	42.43	0.63 (0.595–0.668)^a^	<.0001
Pulm. oedema or cardiogenic shock	956	20.94	786	17.67	1.224 (1.114–1.345)	<.0001
ESKD	1031	22.35	954	21.00	1.085 (0.994–1.185)	.07
Incident AF	269	12.37	243	11.33	1.08 (0.908–1.285)	.38
VT/VF/Cardiac arrest	737	16.27	713	15.98	1.025 (0.925–1.136)	.64
Cardiovascular-related hospitalization	2289	47.01	2806	57.18	0.816 (0.783–0.850)^a^	<.0001

	Sodium Zirconium		Sodium polystyrene sulfonate			
	(*n* = 15 769)		(*n* = 15 769)			
	Number of events	Yearly rate, %	Number of events	Yearly rate, %	Hazard ratio (95% CI)	*P* value
Death	5107	35.88	4691	32.82	1.089 (1.053–1.125)^a^	<.0001
Incident MI	700	9.25	656	8.29	1.132 (1.018–1.260)	.02
Hospitalization for HF	6718	48.08	6042	42.99	1.184 (1.143–1.225)	<.0001
Pulm. oedema or cardiogenic shock	3043	23.70	2387	18.55	1.343 (1.273–1.417)	<.0001
ESKD	3224	24.33	2941	22.05	1.137 (1.082–1.196)	<.0001
Incident AF	650	11.06	666	10.86	1.018 (0.913–1.134)	.75
VT/VF/Cardiac arrest	2153	17.02	2054	15.96	1.08 (1.017–1.148)	.01
Cardiovascular-related hospitalization	8825	63.58	8061	57.92	1.193 (1.157–1.229)	<.0001

	Sodium Zirconium		Patiromer			
	(*n* = 4381)		(*n* = 4381)			
	Number of events	Yearly rate, %	Number of events	Yearly rate, %	Hazard ratio (95% CI)	*P* value
Death	1499	37.52	1568	38.02	0.993 (0.925–1.066)	.85
Incident MI	199	9.24	238	10.45	0.892 (0.739–1.076)	.23
Hospitalization for HF	1853	46.71	830	20.68	2.735 (2.519–2.969)	<.0001
Pulm. oedema or cardiogenic shock	863	23.91	863	23.27	1.057 (0.962–1.161)	.25
ESKD	936	25.07	968	25.25	1.004 (0.918–1.099)	.93
Incident AF	186	11.83	223	12.87	0.893 (0.735–1.085)	.25
VT/VF/Cardiac arrest	628	17.91	609	16.67	1.087 (0.972–1.215)	.14
Cardiovascular-related hospitalization	2455	62.87	1650	42.85	1.488 (1.421–1.558)^a^	<.0001

AF, atrial fibrillation; ESKD, end-stage kidney disease; HF, heart failure; MI, myocardial infarction; VF, ventricular fibrillation; VT, ventricular tachycardia.

^a^Risk ratio is presented instead of hazard ratio since the proportional hazard assumption was violated.

### Sodium zirconium cyclosilicate vs sodium polystyrene sulfonate

In the matched SZC–SPS cohort (*n* = 15 769 per group), SZC was associated with higher risks of all-cause mortality (35.88% vs 32.82%; RR 1.089, 95% CI 1.053–1.125; *P* < .0001), myocardial infarction (HR 1.132, 95% CI 1.018–1.260; *P* = .02), hospitalization for HF (48.08% vs 42.99%; HR 1.184, 95% CI 1.143–1.225; *P* < .0001), pulmonary oedema or cardiogenic shock (HR 1.343, 95% CI 1.273–1.417; *P* < .0001), end-stage kidney disease (HR 1.137, 95% CI 1.082–1.196; *P* < .0001), ventricular tachyarrhythmia or cardiac arrest (HR 1.08, 95% CI 1.017–1.148; *P* = .01), and cardiovascular-related hospitalization (HR 1.193, 95% CI 1.157–1.229; *P* < .0001). There was no significant difference in incident atrial fibrillation (HR 1.018; *P* = .75) (*Table 4*).

### Sodium zirconium cyclosilicate vs patiromer

In the direct comparison of SZC and patiromer (*n* = 4381 per group), there was no significant difference in all-cause mortality (HR 0.993, 95% CI 0.925–1.066; *P* = .85), myocardial infarction (HR 0.892; *P* = .23), incident atrial fibrillation (HR 0.893; *P* = .25), pulmonary oedema or cardiogenic shock (HR 1.057; *P* = .25), end-stage kidney disease (HR 1.004; *P* = .93), or ventricular tachyarrhythmia or cardiac arrest (HR 1.087; *P* = .14). However, SZC was associated with a markedly higher risk of hospitalization for HF (46.7% vs 20.7%; HR 2.735, 95% CI 2.519–2.969; *P* < .0001) and cardiovascular-related hospitalization (62.9% vs 42.9%; RR 1.488, 95% CI 1.421–1.558; *P* < .0001) (*Table 4*).

### Sensitivity analyses

Results were consistent when follow-up was truncated at 6 months, with similar direction and magnitude of associations for HF hospitalization and cardiovascular-related hospitalization across comparisons (Supplementary Table S2). Effect estimates were also consistent in direction over longer follow-up extended up to 48 months, with modest attenuation in magnitude (Supplementary Table S3). In an exploratory analysis, initiation of SZC at 10 g vs 5 g was associated with modestly higher risks of death, myocardial infarction, HF hospitalization, and cardiovascular-related hospitalization (Supplementary Table S4). In patients with HFrEF, sunburst analyses describing post-index GDMT pathways over the first 365 days showed broadly similar patterns of background HF treatment combinations across the three potassium binder groups, including beta-blockers, ACEi/ARB/ARNI, mineralocorticoid receptor antagonists, and SGLT2 inhibitors (Supplementary Figure S1).

### Interaction analysis according to heart failure phenotype

In stratified analyses according to HF phenotype, associations between SZC and patiromer were directionally consistent across HFrEF and HFpEF. However, formal interaction testing demonstrated significant effect modification for cardiovascular-related hospitalization (*P* for interaction = .01) and pulmonary oedema or cardiogenic shock (*P* = .03), with stronger relative associations observed in HFpEF (Supplementary Table S5). A borderline interaction was observed for hospitalization for HF (*P* = .05). No significant interaction by HF subtype was detected for all-cause mortality, incident myocardial infarction, end-stage kidney disease, or incident AF. These findings suggest that the excess risk associated with SZC compared with patiromer may be more pronounced for congestion-related endpoints in HFpEF.

## Discussion

In this large real-world comparative effectiveness study using a target-trial emulation framework, we observed substantial differences in cardiovascular outcomes among patients with HF and hyperkalaemia treated with patiromer, SZC, or SPS. Several key findings emerge. First, compared with SPS, patiromer was associated with a lower risk of hospitalization for HF and cardiovascular-related hospitalization, although a modest increase in all-cause mortality was observed. Second, SZC was associated with higher risks of death, HF hospitalization, myocardial infarction, end-stage kidney disease, and cardiovascular hospitalization compared with SPS. Third, in direct comparison, SZC was associated with markedly higher rates of HF and cardiovascular hospitalization than patiromer, without a significant difference in mortality. The large effect size observed for HF hospitalization in the SZC vs patiromer comparison raises the possibility of residual confounding or channelling bias, despite extensive adjustment. However, these findings raise the possibility that potassium binders may not be interchangeable with respect to cardiovascular outcomes in patients with HF and hyperkalaemia. Given the observational design and the large effect sizes observed in some comparisons, these associations may substantially reflect treatment selection and underlying patient complexity rather than direct pharmacologic toxicity.

Hyperkalaemia is common in HF, particularly in patients with chronic kidney disease, diabetes, and those receiving RAASi.^1,2^ Both hypo- and hyperkalaemia are associated with adverse cardiovascular outcomes in a U-shaped relationship, including arrhythmias and mortality.^1,6^ Importantly, hyperkalaemia frequently leads to down-titration or discontinuation of GDMT, potentially worsening long-term prognosis.^1,7^ Novel potassium binders have therefore been welcomed as tools to maintain normokalaemia and facilitate GDMT optimization.^1,5,8–15^ However, these agents differ in pharmacologic properties, notably in their exchange ions.^4,5,16^ Patiromer exchanges potassium for calcium, whereas SZC exchanges potassium for sodium and hydrogen.^2,5,9,12,17,18^ SPS, an older resin, also relies on sodium exchange and has long been associated with gastrointestinal toxicity and electrolyte disturbances.^1^

The differential outcome profiles observed in our study may plausibly relate, at least in part, to sodium load and its haemodynamic consequences. Each 5 g dose of SZC contains approximately 400 mg of sodium. In patients with HF—particularly those with reduced cardiac reserve, elevated filling pressures, and impaired renal sodium handling—even modest increases in sodium exposure could plausibly worsen congestion. This may increase ventricular filling pressures and precipitate HF decompensation. This mechanism provides biological plausibility for the observed associations with HF hospitalization and cardiovascular hospitalization observed with SZC, particularly in comparison with patiromer, which does not involve sodium exchange. However, the magnitude of the observed effect sizes is unlikely to be fully explained by sodium exposure alone. Alternative explanations, including preferential prescribing in patients with more severe hyperkalaemia, advanced CKD, recurrent congestion, or greater HF severity, likely also contributed to the observed associations. Although our findings suggest a possible dose–response relationship when comparing SZC 5 g vs 10 g, interpretation remains limited by the absence of data on dose titration, treatment duration, and cumulative sodium exposure. As a result, the specific contribution of higher SZC doses to the observed associations cannot be reliably determined. While SPS is also sodium-based, its use is often short-term and variable in dosing, and prescribing patterns may differ substantially from those of newer agents. The consistent signal for increased HF-related outcomes with SZC across multiple comparisons provides support for the hypothesis that sodium exchange may be clinically relevant in this vulnerable population.

Although the risk of HF hospitalization differed substantially between treatment groups, particularly in the direct comparison of SZC and patiromer, this pattern was not consistently mirrored for pulmonary oedema or cardiogenic shock. These endpoints likely capture different clinical spectra. Hospitalization for HF encompasses a broad range of decompensation severities, including progressive congestion requiring inpatient management, whereas pulmonary oedema and cardiogenic shock reflect more acute and severe presentations. The observed dissociation may therefore suggest that the associations may relate more to differences in congestion burden than to increased rates of fulminant haemodynamic collapse.

Notably, interaction analyses suggested that the excess risk associated with SZC compared with patiromer was more pronounced in HFpEF for cardiovascular-related hospitalization and pulmonary oedema or cardiogenic shock. This observation is biologically plausible. Patients with HFpEF are often characterized by greater ventricular stiffness, higher filling pressures, and heightened sensitivity to volume shifts.^19–21^ Even modest increases in sodium exposure may therefore translate into greater congestion burden in this phenotype. While these subgroup findings should be interpreted cautiously, they raise the hypothesis that sodium-exchange binders may differentially affect HF phenotypes according to underlying haemodynamic reserve.

In contrast to the consistent differences observed for HF and cardiovascular hospitalization, we did not detect major differences between treatment groups for incident atrial fibrillation or ventricular tachyarrhythmias. This finding is notable given the established electrophysiologic consequences of dyskalaemia. However, the principal mechanistic distinction between binders relates to sodium exchange rather than potassium-lowering efficacy *per se*. It is therefore plausible that differences in volume status and congestion risk may be more pronounced than differences in arrhythmic susceptibility, particularly if potassium levels were effectively corrected across groups.

These findings raise the possibility that the observed differences may partly relate more to haemodynamic pathways than through differential effects on cardiac electrophysiology. Notably, prior comparative observational studies of potassium binders have largely focused on HF hospitalization and mortality, with limited evaluation of arrhythmic endpoints, making our findings in this domain exploratory.

Our results corroborate and meaningfully expand upon prior observational data. In a claims-based cohort study comparing new users of SZC and patiromer, Zhuo *et al*.^3^ reported a numerically higher, though not statistically significant, risk of HF hospitalization with SZC. That study included approximately 1100 SZC initiators and was not restricted to a HF-specific population. More recently, an electronic health record–based analysis by Desai *et al*.^4^ reported significantly higher risks of HF hospitalization, major oedema, and death among SZC users compared with patiromer. The magnitude and direction of associations in our study are consistent with these signals. Our study extends the evidence base in several important ways: we focused specifically on patients with HF and hyperkalaemia, included a substantially larger matched population, evaluated a broader range of cardiovascular endpoints—including myocardial infarction and end-stage kidney disease—and incorporated a direct comparison with SPS. The convergence of findings across independent data sources strengthens the credibility of a potential association between SZC use and fluid-related or cardiovascular outcomes in routine practice.

These findings should be interpreted in the context of randomized trials of potassium binders. Such trials have consistently demonstrated effective potassium lowering and overall neutral safety profiles, but were not designed or powered to assess hard cardiovascular outcomes in high-risk HF populations.^1,5^ For example, trials of SZC and patiromer have demonstrated effective potassium reduction and facilitation of RAASi therapy but were not powered to detect differences in HF hospitalization or mortality.^10,15,17,22,23^ In addition, emergency-use studies of SZC have suggested reductions in urgent dialysis requirements in acute hyperkalaemia settings;^17^ however, acute potassium correction in hospitalized patients differs fundamentally from chronic outpatient management in HF populations.^1^ The discordance with randomized trials likely reflects differences in design, patient selection, and endpoints, with trials focusing on potassium control rather than cardiovascular outcomes. Our findings suggest that efficacy in lowering potassium does not necessarily translate into neutral cardiovascular effects, particularly when differences in exchange ions and sodium exposure are considered. In extended follow-up analyses, effect estimates remained consistent in direction, with modest attenuation in magnitude. These findings do not support the presence of a delayed compensatory benefit, although such effects cannot be definitively excluded given the observational design. Given the potential residual confounding, this warrants confirmation in dedicated randomized trials.

The modestly higher mortality observed with patiromer compared with SPS should be interpreted cautiously. It may reflect residual confounding or channelling bias, with preferential use in more fragile or clinically complex patients rather than a causal treatment effect. In contrast, direct comparison between SZC and patiromer did not reveal a significant mortality difference despite marked differences in HF hospitalization risk. These patterns may reflect residual confounding, differences in patient selection, competing risks, or unmeasured clinical characteristics influencing prescribing decisions. Although extensive propensity score matching across more than 70 baseline variables achieved excellent covariate balance, observational analyses cannot eliminate all sources of bias. Therefore, mortality findings should be viewed as hypothesis-generating rather than definitive.

From a clinical perspective, these data suggest that potassium binder selection in HF may warrant individualized consideration beyond potassium control alone. While maintaining normokalaemia to preserve GDMT remains a central therapeutic goal,^1,15^ the potential haemodynamic consequences of sodium-containing binders merit attention, particularly in patients with advanced HF, recurrent congestion, or high diuretic requirements.^4^ The strategy of enabling RAASi continuation through potassium lowering may involve trade-offs if the potassium-lowering agent itself influences volume status.^1,4^ Our findings do not negate the utility of SZC but suggest that careful patient selection and monitoring may be appropriate, especially in congestion-prone phenotypes. The lack of reliable longitudinal data on GDMT optimization, including treatment persistence and dose titration, precludes assessment of whether differences in outcomes reflect direct drug effects or downstream differences in HF therapy.

This study has several strengths. We leveraged a large, multinational real-world database encompassing diverse healthcare settings. The target-trial emulation framework explicitly defined eligibility criteria, exposure assignment, start of follow-up, and outcome definitions to approximate a randomized comparison. Active-comparator designs and extensive propensity score matching across many clinical and laboratory variables reduced confounding. Sensitivity analyses truncating follow-up at six months yielded consistent results, suggesting that associations were not driven solely by late events. Furthermore, exclusion of patients with dialysis or renal transplantation focused the analysis on non–end-stage kidney disease populations most relevant to contemporary HF management.

Nonetheless, limitations must be acknowledged. First, the observational design precludes causal inference. Residual confounding and channelling bias remain possible, particularly if sicker or more volume-sensitive patients were preferentially prescribed one agent over another. This limitation is particularly relevant when interpreting large effect sizes in observational comparative effectiveness analyses. Second, detailed information on dosing, adherence, and cumulative sodium exposure was not available. Exposure was defined at treatment initiation without accounting for adherence or duration, and the lack of reliable time-updated data precluded time-varying analyses. This limitation is particularly relevant for agents such as SPS, which are often used short-term. Although exposure groups were defined at treatment initiation and follow-up began after index treatment assignment, residual bias related to treatment persistence and evolving exposure during follow-up cannot be excluded. Third, dynamic changes in potassium levels and longitudinal RAASi optimization could not be fully captured. The lack of robust longitudinal data on GDMT optimization, including maintenance or up-titration of RAASi and MRA therapies, precludes assessment of whether differences in outcomes reflect direct treatment associations. Alternatively, these differences may reflect downstream effects related to differential GDMT exposure. Fourth, HF phenotype (e.g. reduced vs preserved ejection fraction) was not systematically stratified, and differences in underlying HF biology may influence susceptibility to sodium-related effects. HF phenotype classification relied primarily on administrative coding, as LVEF data were incompletely available, which may have limited more granular phenotypic stratification. Although outcomes were defined using standardized coding and validated approaches within the TriNetX platform, misclassification cannot be entirely excluded. Also, competing risks were not explicitly modelled. Standard Cox models were used to evaluate comparative associations over time; however, in the context of substantial mortality, competing death may influence estimates for non-fatal congestion-related outcomes such as HF hospitalization and cardiovascular hospitalization. Finally, the study may not be powered to detect modest effects or subgroup interactions, and causal inference cannot be established. These findings should therefore be interpreted as hypothesis-generating.

Future research should aim to clarify whether the associations observed reflect potential sodium-mediated effects, differential patient selection, or other mechanisms. Randomized head-to-head trials comparing SZC and patiromer in HF populations, powered for hard cardiovascular endpoints such as HF hospitalization and cardiovascular death, would provide definitive evidence. Mechanistic studies incorporating biomarkers of congestion, natriuretic peptides, and haemodynamic assessments may further elucidate the relationship between sodium-exchange binders and volume status. Precision approaches identifying HF phenotypes most vulnerable to sodium load may help tailor potassium binder selection in clinical practice.

## Conclusions

In patients with HF and hyperkalaemia, potassium binders demonstrated distinct cardiovascular outcome profiles in routine clinical practice. SZC was associated with higher risks of HF and cardiovascular hospitalization compared with both patiromer and SPS, while patiromer was associated with lower HF hospitalization compared with SPS. These findings should be interpreted cautiously and considered hypothesis-generating but raise the possibility that potassium binders may differ in cardiovascular risk profiles. They underscore the need for randomized trials specifically powered for hard cardiovascular endpoints to guide optimal therapeutic selection in HF.

## Supplementary Material

xvag248_Supplementary_Data

## Declarations

### Data Availability

No data were generated or analysed for this manuscript.

### Pre-registered Clinical Trial Number

None supplied.
