# Supplementary material for: Cardiovascular outcomes with potassium binders in patients with heart failure and hyperkalaemia
Source: ESC Heart Fail. 2026 Sep 28;13(5):xvag248. doi: 10.1093/eschf/xvag248 (PMC13639487; doi:10.1093/eschf/xvag248)
Supplement: Supplementary file 1 [file xvag248_supplementary_data.docx]

# Supplemental Table 1. Codes used in the TriNetX analysis

## Diseases and comorbidities (ICD-10-CM)

|  | ***Codes*** |
| --- | --- |
| Hypertension | I10 |
| Diabetes mellitus | E08–E13 |
| Dyslipidaemia | E78 |
| Obesity | E65–E68 |
| Heart failure | I50 |
| Coronary artery disease / ischemic heart disease | I20–I25 |
| Myocardial infarction | I21, I22 |
| Atrial fibrillation or flutter | I48 |
| Atrial flutter (typical/atypical) | I48.3, I48.4, I48.92 |
| Ischemic stroke | I63 |
| Systemic or arterial thromboembolism | I74 |
| Intracranial haemorrhage | I61 |
| Chronic kidney disease / acute kidney failure | N17–N19 |
| Chronic obstructive pulmonary disease | J44 |
| Sleep apnoea | G47.3 |
| Peripheral vascular disease | I73.9 |
| Valvular heart disease | I34, I35 |
| Conduction disorders | I44, I45 |
| Alcohol abuse | F10 |
| Neoplasms | C00–D49 |
| Anaemia | D64.9 |
| Pacemaker or ICD in situ | Z95.0, Z95.810 |
| Long-term anticoagulant use | Z79.01 |

## Medications (ATC)

|  | ***Codes*** |
| --- | --- |
| Beta-blockers | C07 |
| ACE inhibitors | C09A |
| Angiotensin-II receptor blockers | C09C |
| Diuretics | C03 |
| Calcium channel blockers | C08 |
| Antiarrhythmic drugs | C01B |
| Amiodarone | C01BD01 |
| Anticoagulants | B01A |
| Direct oral anticoagulants | B01AE07, B01AF01–03 |
| Statins | C10AA |
| Antidiabetic drugs | A10 |

**Supplemental Table 2.** Clinical outcomes during FU in the 6 first months in the matched populations (follow-up 0.4 ± 0.2 years, median 0.5, IQR 0.3)

|  | **Patiromer** | | **Sodium polystyrene sulfonate** | |  |  |
| --- | --- | --- | --- | --- | --- | --- |
|  | **Number of events** | **Yearly rate, %** | **Number of events** | **Yearly rate, %** | **Hazard ratio (95% CI)** | **p value** |
| **Death** | 1624 | 60.09 | 1499 | 56.26 | 1.09 (1.016-1.169) | 0.02 |
| **Incident MI** | 185 | 11.39 | 173 | 10.81 | 1.066 (0.867-1.312) | 0.54 |
| **Hospitalization for HF** | 1485 | 55.99 | 2211 | 84.60 | 0.672 (0.636-0.710) * | <0.0001 |
| **Pulm. oedema or cardiogenic shock** | 844 | 33.75 | 677 | 27.53 | 1.252 (1.131-1.385) | <0.0001 |
| **ESKD** | 934 | 36.59 | 864 | 34.38 | 1.086 (0.990-1.191) | 0.08 |
| **Incident AF** | 188 | 15.51 | 177 | 14.98 | 1.055 (0.860-1.296) | 0.61 |
| **VT/VF/Cardiac arrest** | 654 | 26.20 | 630 | 25.55 | 1.03 (0.923-1.149) | 0.6 |
| **Cardiovascular-related hospitalization** | 2339 | 89.17 | 2852 | 109.39 | 0.751 (0.711-0.794) | <0.0001 |

|  | **Sodium Zirconium** | | **Sodium polystyrene sulfonate** | |  |  |
| --- | --- | --- | --- | --- | --- | --- |
|  | **Number of events** | **Yearly rate, %** | **Number of events** | **Yearly rate, %** | **Hazard ratio (95% CI)** | **p value** |
| **Death** | 4289 | 59.64 | 3807 | 52.95 | 1.127 (1.085-1.170) * | <0.0001 |
| **Incident MI** | 488 | 11.86 | 441 | 10.39 | 1.154 (1.014-1.312) | 0.03 |
| **Hospitalization for HF** | 6490 | 92.29 | 5822 | 82.64 | 1.181 (1.139-1.223) | <0.0001 |
| **Pulm. oedema or cardiogenic shock** | 2578 | 38.30 | 1994 | 29.79 | 1.293 (1.225-1.365) * | <0.0001 |
| **ESKD** | 2907 | 42.48 | 2592 | 37.67 | 1.154 (1.094-1.217) | <0.0001 |
| **Incident AF** | 460 | 14.67 | 458 | 14.14 | 1.033 (0.907-1.175) | 0.63 |
| **VT/VF/Cardiac arrest** | 1804 | 27.10 | 1701 | 25.40 | 1.083 (1.014-1.157) | 0.02 |
| **Cardiovascular-related hospitalization** | 8428 | 119.80 | 7635 | 108.64 | 1.104 (1.080-1.128) * | <0.0001 |

|  | **Sodium Zirconium** | | **Patiromer** | |  |  |
| --- | --- | --- | --- | --- | --- | --- |
|  | **Number of events** | **Yearly rate, %** | **Number of events** | **Yearly rate, %** | **Hazard ratio (95% CI)** | **p value** |
| **Death** | 587 | 54.09 | 565 | 51.44 | 1.07 (0.953-1.201) | 0.25 |
| **Incident MI** | 79 | 12.14 | 74 | 10.90 | 1.115 (0.812-1.532) | 0.5 |
| **Hospitalization for HF** | 810 | 76.29 | 576 | 54.46 | 1.406 (1.285-1.540) * | <0.0001 |
| **Pulm. oedema or cardiogenic shock** | 359 | 34.77 | 253 | 25.20 | 1.419 (1.221-1.650) * | <0.0001 |
| **ESKD** | 486 | 47.18 | 482 | 45.97 | 1.023 (0.902-1.160) | 0.72 |
| **Incident AF** | 67 | 13.18 | 93 | 17.81 | 0.73 (0.533-0.999) | 0.05 |
| **VT/VF/Cardiac arrest** | 223 | 22.32 | 228 | 22.73 | 1.008 (0.838-1.212) | 0.94 |
| **Cardiovascular-related hospitalization** | 1100 | 104.09 | 868 | 82.66 | 1.267 (1.183-1.357) * | <0.0001 |

AF = atrial fibrillation; ESKD = end-stage kidney disease; HF = heart failure; MI = myocardial infarction; VF = ventricular fibrillation; VT = ventricular tachycardia.

* Risk ratio is presented instead of hazard ratio since the proportional hazard assumption was violated.

**Supplemental Table 3.** Clinical outcomes during FU in the matched populations with follow-up extended up to 48 months (follow-up 1.7 ± 1.5 years, median 1.3, IQR 3)

|  | **Patiromer** | | **Sodium polystyrene sulfonate** | |  |  |
| --- | --- | --- | --- | --- | --- | --- |
|  | **Number of events** | **Yearly rate, %** | **Number of events** | **Yearly rate, %** | **Hazard ratio (95% CI)** | **p value** |
| **Death** | 2719 | 15.2 | 2584 | 14.4 | 1.089 (1.032-1.149) | 0.002 |
| **Incident MI** | 507 | 6.4 | 501 | 6.0 | 1.059 (0.936-1.199) | 0.36 |
| **Hospitalization for HF** | 1415 | 7.1 | 2413 | 12.4 | 0.586 (0.555-0.619)* | <0.0001 |
| **Pulm. edema or cardiogenic shock** | 1339 | 9.3 | 1196 | 8.4 | 1.159 (1.072-1.253) | <0.0001 |
| **ESKD** | 1365 | 9.0 | 1308 | 8.6 | 1.058 (0.981-1.141) | 0.14 |
| **Incident AF** | 476 | 7.2 | 468 | 7.3 | 1.04 (0.915-1.181) | 0.55 |
| **VT/VF/Cardiac arrest** | 1020 | 7.2 | 1015 | 7.3 | 1.025 (0.940-1.118) | 0.58 |
| **Cardiovascular-related hospitalization** | 2656 | 15.5 | 3289 | 18.0 | 0.808 (0.779-0.837)* | <0.0001 |

|  | **Sodium Zirconium** | | **Sodium polystyrene sulfonate** | |  |  |
| --- | --- | --- | --- | --- | --- | --- |
|  | **Number of events** | **Yearly rate, %** | **Number of events** | **Yearly rate, %** | **Hazard ratio (95% CI)** | **p value** |
| **Death** | 7352 | 14.6 | 7738 | 14.1 | 0.95 (0.928-0.973)* | <0.0001 |
| **Incident MI** | 1358 | 6.7 | 1429 | 5.8 | 1.175 (1.090-1.266) | <0.0001 |
| **Hospitalization for HF** | 8239 | 14.1 | 7826 | 13.4 | 1.053 (1.029-1.077)* | <0.0001 |
| **Pulm. edema or cardiogenic shock** | 4251 | 10.2 | 3713 | 8.5 | 1.145 (1.102-1.190)* | <0.0001 |
| **ESKD** | 3899 | 8.9 | 3801 | 8.0 | 1.13 (1.081-1.182) | <0.0001 |
| **Incident AF** | 1246 | 7.5 | 1352 | 6.9 | 1.088 (1.006-1.175) | 0.03 |
| **VT/VF/Cardiac arrest** | 3082 | 8.0 | 3117 | 7.0 | 0.989 (0.945-1.034)* | 0.62 |
| **Cardiovascular-related hospitalization** | 10811 | 19.3 | 10387 | 18.4 | 1.041 (1.024-1.058)* | <0.0001 |

|  | **Sodium Zirconium** | | **Patiromer** | |  |  |
| --- | --- | --- | --- | --- | --- | --- |
|  | **Number of events** | **Yearly rate, %** | **Number of events** | **Yearly rate, %** | **Hazard ratio (95% CI)** | **p value** |
| **Death** | 2546 | 15.0 | 2837 | 15.2 | 0.984 (0.932-1.038) | 0.55 |
| **Incident MI** | 519 | 6.9 | 530 | 6.5 | 1.161 (1.028-1.312) | 0.02 |
| **Hospitalization for HF** | 2578 | 12.7 | 1430 | 6.9 | 1.803 (1.709-1.902)* | <0.0001 |
| **Pulm. edema or cardiogenic shock** | 1497 | 10.6 | 1425 | 9.5 | 1.166 (1.084-1.254) | <0.0001 |
| **ESKD** | 1381 | 9.2 | 1419 | 9.0 | 0.973 (0.912-1.038)* | 0.41 |
| **Incident AF** | 450 | 7.6 | 487 | 7.2 | 1.059 (0.931-1.204) | 0.38 |
| **VT/VF/Cardiac arrest** | 1066 | 8.2 | 1085 | 7.3 | 0.982 (0.910-1.061)* | 0.65 |
| **Cardiovascular-related hospitalization** | 3499 | 18.5 | 2741 | 15.5 | 1.277 (1.233-1.322)* | <0.0001 |

AF = atrial fibrillation; ESKD = end-stage kidney disease; HF = heart failure; MI = myocardial infarction; VF = ventricular fibrillation; VT = ventricular tachycardia.

* Risk ratio is presented instead of hazard ratio since the proportional hazard assumption was violated.

**Supplemental Table 4.** Clinical outcomes during FU in the matched populations of HF patients treated with Sodium Zirconium 5 or 10 g (follow-up 0.7 ± 0.4 years, median 1, IQR 0.8)

|  | **Sodium Zirconium 10g** | | **Sodium Zirconium 5g** | |  |  |
| --- | --- | --- | --- | --- | --- | --- |
|  | **(n = 7127)** | | **(n = 7127)** | |  |  |
|  | **Number of events** | **Yearly rate, %** | **Number of events** | **Yearly rate, %** | **Hazard ratio (95% CI)** | **p value** |
| **Death** | 2412 | 36.8 | 2294 | 35.3 | 1.061 (1.002-1.124) | 0.04 |
| **Incident MI** | 349 | 9.7 | 306 | 8.5 | 1.171 (1.005-1.366) | 0.04 |
| **Hospitalization for HF** | 3920 | 60.8 | 3688 | 56.7 | 1.063 (1.031-1.096)* | <0.0001 |
| **Pulm. edema or cardiogenic shock** | 1403 | 23.1 | 1358 | 22.4 | 1.035 (0.960-1.115) | 0.36 |
| **ESKD** | 1556 | 25.3 | 1476 | 23.7 | 1.052 (0.980-1.130) | 0.15 |
| **Incident AF** | 272 | 10.1 | 262 | 9.9 | 1.054 (0.890-1.249) | 0.54 |
| **VT/VF/Cardiac arrest** | 1040 | 17.7 | 1175 | 19.4 | 0.885 (0.820-0.956)* | 0.002 |
| **Cardiovascular-related hospitalization** | 4811 | 74.4 | 4630 | 71.1 | 1.039 (1.015-1.064)* | 0.001 |

AF = atrial fibrillation; ESKD = end-stage kidney disease; HF = heart failure; MI = myocardial infarction; VF = ventricular fibrillation; VT = ventricular tachycardia.

* Risk ratio is presented instead of hazard ratio since the proportional hazard assumption was violated.

**Supplemental Table 5.** Interaction analysis for clinical outcomes for HF patients treated with Sodium Zirconium or Patiromer according to HFrEF or HFpEF

|  |  | **Sodium Zirconium** | | | **Patiromer** | | |  |  |
| --- | --- | --- | --- | --- | --- | --- | --- | --- | --- |
|  |  | **(n = 5144)** | | | **(n = 5144)** | | |  |  |
|  |  | **Number of patients** | **Number of events** | **Yearly rate, %** | **Number of  patients** | **Number of events** | **Yearly rate, %** | **Hazard ratio  (95% CI)** | **p value for interaction** |
| **Death** | **HFrEF** | 2335 | 796 | 37.57 | 2335 | 833 | 38.57 | 0.977 (0.886-1.076) |  |
|  | **HFpEF** | 2809 | 935 | 36.19 | 2809 | 956 | 36.95 | 0.992 (0.906-1.085) | 0.82 |
| **Incident MI** | **HFrEF** | 2335 | 112 | 10.67 | 2335 | 86 | 7.92 | 1.411 (1.066-1.869) |  |
|  | **HFpEF** | 2809 | 160 | 10.52 | 2809 | 134 | 9.06 | 1.231 (0.979-1.549) | 0.46 |
| **Hospitalization for HF** | **HFrEF** | 2335 | 1132 | 53.19 | 2335 | 676 | 31.80 | 2.002 (1.820-2.202) |  |
|  | **HFpEF** | 2809 | 1287 | 50.68 | 2809 | 664 | 25.71 | 2.291 (2.086-2.516) | 0.05 |
| **Pulm. oedema or cardiogenic shock** | **HFrEF** | 2335 | 526 | 26.87 | 2335 | 504 | 25.48 | 1.071 (0.948-1.211) |  |
|  | **HFpEF** | 2809 | 567 | 24.58 | 2809 | 458 | 20.05 | 1.293 (1.144-1.463) | 0.03 |
| **ESKD** | **HFrEF** | 2335 | 397 | 20.00 | 2335 | 399 | 20.44 | 1.014 (0.883-1.165) |  |
|  | **HFpEF** | 2809 | 604 | 25.23 | 2809 | 557 | 23.80 | 1.127 (1.004-1.264) | 0.25 |
| **Incident AF** | **HFrEF** | 2335 | 108 | 13.30 | 2335 | 101 | 12.07 | 1.089 (0.830-1.428) |  |
|  | **HFpEF** | 2809 | 136 | 12.89 | 2809 | 135 | 12.41 | 1.046 (0.825-1.328) | 0.83 |
| **VT/VF/Cardiac arrest** | **HFrEF** | 2335 | 426 | 22.21 | 2335 | 470 | 24.19 | 0.915 (0.802-1.043) |  |
|  | **HFpEF** | 2809 | 293 | 12.95 | 2809 | 266 | 11.72 | 1.12 (0.949-1.322) | 0.06 |
| **Cardiovascular-related hospitalization** | **HFrEF** | 2335 | 1443 | 68.33 | 2335 | 1095 | 52.83 | 1.633 (1.510-1.767) |  |
|  | **HFpEF** | 2809 | 1699 | 67.56 | 2809 | 1133 | 46.00 | 1.885 (1.748-2.033) | 0.01 |

AF = atrial fibrillation; ESKD = end-stage kidney disease; HF = heart failure; HFpEF = heart failure with preserved ejection fraction; HFrEF = heart failure with reduced ejection fraction; MI = myocardial infarction; VF = ventricular fibrillation; VT = ventricular tachycardia.

**Supplemental Table 3.** Clinical outcomes during FU in the matched populations (follow-up 1.7 ± 1.5 years, median 1.3, IQR 3)

|  | **Patiromer** | | **Sodium polystyrene sulfonate** | |  |  |
| --- | --- | --- | --- | --- | --- | --- |
|  | **(n = 5689)** | | **(n = 5689)** | |  |  |
|  | **Number of events** | **Yearly rate, %** | **Number of events** | **Yearly rate, %** | **Hazard ratio (95% CI)** | **p value** |
| **Death** | 2719 | 15.2 | 2584 | 14.4 | 1.089 (1.032-1.149) | 0.002 |
| **Incident MI** | 507 | 6.4 | 501 | 6.0 | 1.059 (0.936-1.199) | 0.36 |
| **Hospitalization for HF** | 1415 | 7.1 | 2413 | 12.4 | 0.586 (0.555-0.619)* | <0.0001 |
| **Pulm. edema or cardiogenic shock** | 1339 | 9.3 | 1196 | 8.4 | 1.159 (1.072-1.253) | <0.0001 |
| **ESKD** | 1365 | 9.0 | 1308 | 8.6 | 1.058 (0.981-1.141) | 0.14 |
| **Incident AF** | 476 | 7.2 | 468 | 7.3 | 1.04 (0.915-1.181) | 0.55 |
| **VT/VF/Cardiac arrest** | 1020 | 7.2 | 1015 | 7.3 | 1.025 (0.940-1.118) | 0.58 |
| **Cardiovascular-related hospitalization** | 2656 | 15.5 | 3289 | 18.0 | 0.808 (0.779-0.837)* | <0.0001 |

|  | **Sodium Zirconium** | | **Sodium polystyrene sulfonate** | |  |  |
| --- | --- | --- | --- | --- | --- | --- |
|  | **(n = 17183)** | | **(n = 17183)** | |  |  |
|  | **Number of events** | **Yearly rate, %** | **Number of events** | **Yearly rate, %** | **Hazard ratio (95% CI)** | **p value** |
| **Death** | 7352 | 14.6 | 7738 | 14.1 | 0.95 (0.928-0.973)* | <0.0001 |
| **Incident MI** | 1358 | 6.7 | 1429 | 5.8 | 1.175 (1.090-1.266) | <0.0001 |
| **Hospitalization for HF** | 8239 | 14.1 | 7826 | 13.4 | 1.053 (1.029-1.077)* | <0.0001 |
| **Pulm. edema or cardiogenic shock** | 4251 | 10.2 | 3713 | 8.5 | 1.145 (1.102-1.190)* | <0.0001 |
| **ESKD** | 3899 | 8.9 | 3801 | 8.0 | 1.13 (1.081-1.182) | <0.0001 |
| **Incident AF** | 1246 | 7.5 | 1352 | 6.9 | 1.088 (1.006-1.175) | 0.03 |
| **VT/VF/Cardiac arrest** | 3082 | 8.0 | 3117 | 7.0 | 0.989 (0.945-1.034)* | 0.62 |
| **Cardiovascular-related hospitalization** | 10811 | 19.3 | 10387 | 18.4 | 1.041 (1.024-1.058)* | <0.0001 |

|  | **Sodium Zirconium** | | **Patiromer** | |  |  |
| --- | --- | --- | --- | --- | --- | --- |
|  | **(n = 5904)** | | **(n = 5904)** | |  |  |
|  | **Number of events** | **Yearly rate, %** | **Number of events** | **Yearly rate, %** | **Hazard ratio (95% CI)** | **p value** |
| **Death** | 2546 | 15.0 | 2837 | 15.2 | 0.984 (0.932-1.038) | 0.55 |
| **Incident MI** | 519 | 6.9 | 530 | 6.5 | 1.161 (1.028-1.312) | 0.02 |
| **Hospitalization for HF** | 2578 | 12.7 | 1430 | 6.9 | 1.803 (1.709-1.902)* | <0.0001 |
| **Pulm. edema or cardiogenic shock** | 1497 | 10.6 | 1425 | 9.5 | 1.166 (1.084-1.254) | <0.0001 |
| **ESKD** | 1381 | 9.2 | 1419 | 9.0 | 0.973 (0.912-1.038)* | 0.41 |
| **Incident AF** | 450 | 7.6 | 487 | 7.2 | 1.059 (0.931-1.204) | 0.38 |
| **VT/VF/Cardiac arrest** | 1066 | 8.2 | 1085 | 7.3 | 0.982 (0.910-1.061)* | 0.65 |
| **Cardiovascular-related hospitalization** | 3499 | 18.5 | 2741 | 15.5 | 1.277 (1.233-1.322)* | <0.0001 |

AF = atrial fibrillation; ESKD = end-stage kidney disease; HF = heart failure; MI = myocardial infarction; VF = ventricular fibrillation; VT = ventricular tachycardia.

* Risk ratio is presented instead of hazard ratio since the proportional hazard assumption was violated.

**Supplemental Figure 1.** Sunburst diagrams depicting post-index patterns of guideline-directed medical therapy (GDMT) combinations during the first 365 days following initiation of patiromer, sodium zirconium cyclosilicate (SZC), or sodium polystyrene sulfonate (SPS) in patients with HFrEF and hyperkalaemia. Treatment pathways were constructed using combinations of beta-blockers, angiotensin-converting enzyme inhibitors/angiotensin receptor blockers/angiotensin receptor–neprilysin inhibitors (ACEi/ARB/ARNI), mineralocorticoid receptor antagonists (MRA), and sodium–glucose cotransporter-2 inhibitors (SGLT2i). Inner segments represent the most frequent treatment combinations, whereas outer segments depict subsequent or less frequent pathways over follow-up. A treatment line included any treatment combination maintained within 90 days.
